# Influence of Residence Area and Basic Livelihood Conditions on the Prevalence and Diagnosis Experience of Osteoporosis in Postmenopausal Women Aged over 50 Years: Evaluation Using Korea National Health and Nutrition Examination Survey Data

**DOI:** 10.3390/ijerph18189478

**Published:** 2021-09-08

**Authors:** Suk-Woong Kang, Ji-Hee Yang, Won-Chul Shin, Yoon-Ji Kim, Min-Hyeok Choi

**Affiliations:** 1 Department of Orthopedic Surgery, Pusan National University Yangsan Hospital, Yangsan 50612, Korea; osksw98@pusan.ac.kr (S.-W.K.); dreami3e5t@pusan.ac.kr (W.-C.S.); 2 Biomedical Research Institute, Pusan National University Yangsan Hospital, Yangsan 50612, Korea; 3 Department of Orthopedic Surgery, Medical College, Pusan National University, Yangsan 50612, Korea; 4 Department of Medicine, Medical College, Pusan National University, Yangsan 50612, Korea; zzzwlgml159@pusan.ac.kr; 5 Department of Preventive and Occupational & Environmental Medicine, Medical College, Pusan National University, Yangsan 50612, Korea; harrypotter79@pusan.ac.kr; 6 Office of Public Healthcare Service, Pusan National University Yangsan Hospital, Yangsan 50612, Korea

**Keywords:** osteoporosis, basic livelihood beneficiaries, health inequality, residence area

## Abstract

Patients with osteoporosis are asymptomatic and are at risk for fractures. Therefore, early detection and interventions are important. We found that a population with a low socioeconomic status living in rural areas was reported to have a high osteoporosis prevalence but a relatively low diagnosis rate. Research on the disparity of osteoporosis prevalence and treatment from the socioeconomic perspective was conducted. This study aimed to investigate the influence of residence area and basic livelihood conditions on osteoporosis prevalence and diagnosis in postmenopausal women aged over 50 years. The cross-sectional data of 1477 postmenopausal women aged over 50 years obtained from the Korea National Health and Nutrition Examination Survey V-2 were analyzed. Univariate analyses were performed to calculate the prevalence and diagnosis rate according to risk factor categories. A multivariate logistic regression analysis was performed to identify the influence of residence area and basic livelihood conditions after controlling for other factors. The osteoporosis prevalence in basic livelihood beneficiaries (53.7%) and rural area residents (41.9%) was higher than that in non-beneficiaries (33.1%) and urban area residents (31.8%). There was no significant difference in the diagnosis rates in relation to the basic livelihood conditions or residence areas. The adjusted odds ratio for the prevalence among the beneficiaries living in rural areas was 2.08 (95% confidence interval: 1.06–4.10). However, the odds ratio for diagnosis was not significantly different. Earlier screening examination policies for osteoporosis in postmenopausal women with a low socioeconomic status living in rural areas are needed.

## 1. Introduction

Osteoporosis is a systemic skeletal disease characterized by low bone quality owing to various causes [1,2]. The prevalence of osteoporosis in women aged >50 years has been reported to be 35.5% in Korea [3]. In addition, the prevalence of osteoporosis in women aged 50–79 years has been reported to be approximately 38% in China and 31.0% in Japan [4,5].

Many countries strive to achieve early detection. However, since osteoporosis is asymptomatic, many patients are not recognized or diagnosed until osteoporosis-related trauma, such as fracture, occurs [6,7,8]. In our previous study, the prevalence rate of osteoporosis was 34.8% in women aged over 50 years, and the diagnosis rate was as low as 22.1% [9]. Therefore, it is important to prevent osteoporosis and facilitate early diagnosis. Osteoporosis is asymptomatic and can only be recognized after a fracture occurs. If a hip or vertebral fracture occurs, the social costs are increased, and a great amount of effort must be put into early detection and treatment of osteoporosis [10].

Osteoporosis has various causative factors, such as age, sex, smoking, alcohol consumption, and systemic disease [2,11]. In addition, it is reported that socioeconomic factors at the individual level, such as educational level and income, are also indirect causes [12,13]. In our previous study, the difference between the cause of osteoporosis and the diagnosis at the socioeconomic level was reported [9].

In this respect, studies on osteoporosis between urban and rural areas have also been conducted [14,15]. According to Lai et al., there are more patients with undertreated osteoporosis in rural areas than in urban areas [16]. Moreover, the quality-adjusted life expectancy (QALE) was reported to be unequal according to income, especially in rural areas [17]. Many diseases, such as cancer, diabetes, kidney disease, and arthritis, are more prevalent in rural areas than in urban areas, and the accessibility and cure rates of diseases have decreased [18,19,20]. Moreover, the disparity between urban and rural areas in health screening and cancer screening rates has been reported [21,22]. In these studies, it was explained that the reason for the disparity between urban and rural areas may be due to differences in medical access due to differences in age structure, socioeconomic level, and distribution of medical resources. 

In Korea, population groups with an income of 30% or less of the median income of that year are considered as having a basic livelihood, and a system for resolving various inequalities is being established. Each year, the basic income line is different, and the proportion of beneficiaries of the national basic livelihood security system is generally maintained at 2–3% of the total population [23]. Since basic living recipients experience many diseases and inequalities, various policies are in place to compensate for such. Medical expenses are also partially reduced; however, some uninsured medical expenses or screening tests that are not included in the mandatory examination items are less accessible owing to cost problems [24,25]. In particular, the population of basic living recipients in rural areas is larger than that in cities, and access to medical care is inferior owing to low income level.

Therefore, the purpose of this study was to: (1) calculate the prevalence and diagnosis rates of osteoporosis in menopausal women aged ≥50 years and compare them according to urban and rural areas; (2) determine the difference between the prevalence and diagnosis rate of osteoporosis among basic livelihood beneficiaries living in urban and rural areas; (3) analyze various socioeconomic factors (focusing on basic livelihood conditions) that affect osteoporosis; and (4) investigate the implications for osteoporosis management policies based on the evidence obtained. 

## 2. Materials and Methods

### 2.1. Data and Study Population

The Korea National Health and Nutrition Examination Survey (KNHANES) was used in this study. It is conducted annually by the Korea Disease Control and Prevention Agency. Every year, approximately 8000 subjects are sampled to represent citizens over the age of one residing in 192 districts in Korea. Screening examinations for chronic diseases and interview surveys on health behaviors, food intake, and socioeconomic status are conducted among the participants of the KNHANES. The data from this survey were used to produce national official indices of health level, health behavior, food and nutrition intake, and prevalence of chronic diseases in Korea. The findings are used as the basis for establishing and implementing national health policies, such as the National Health Promotion Plan [26]. The KNHANES dataset is one of the largest and most representative datasets for research on the prevalence and risk factors of osteoporosis in the country. In our study, the data of 1476 postmenopausal women aged over 50 years who had undergone bone mineral density (BMD) testing among the participants of the KNHANES V-2 were used. The study was approved by the Institutional Review Board of Pusan National University Hospital (No. H-1901-019-075).

### 2.2. Variables

The outcome variables in this study were the prevalence and diagnosis of osteoporosis. Subjects with osteoporosis were considered as the basis of the T-score, as cases with a T-score of ≤−2.5. The T-score is most commonly used to identify osteoporosis and determine the fracture risk [27,28]. T-scores from the KNHANES data were obtained from the BMD measurements of the total femur, femoral neck, and spine using dual-energy X-ray absorptiometry (Hologic Discovery, Hologic, Marlborough, MA, USA). The precision of the equipment was evaluated by scanning 30 randomly selected subjects twice on the same day. The coefficient of variation of the equipment was 1.8% for the total femur, 2.5% for the femoral neck, and 1.9% for the spine. The diagnosis experience of osteoporosis was based on a “Yes” response to the question “Have you ever been diagnosed with osteoporosis by a medical doctor?” in the interview. The major socioeconomic variables in this study were basic livelihood conditions (non-beneficiaries or beneficiaries) and residence areas (urban or rural). Basic livelihood recipients belong to the groups with the lowest socioeconomic status in the country. The basic livelihood condition is useful as a representative indicator of socioeconomic status [25,29,30]. As for the residential area, “dong” was classified as urban, and “eup” and “myeon” were classified as rural. This category is mainly used in research to establish the health disparity between urban and rural areas and the impact of areas on health [31,32].

The general factors associated with the prevalence and diagnosis of osteoporosis were included as independent variables after reviewing studies that were previously reported for osteoporosis risk factors [31,32,33]. The independent factors of osteoporosis included age (50–59 years, 60–69 years, and ≥70 years), educational level (middle school or below and high school or above), physical activity (vigorous physical activity for ≥20 min 3 days a week or moderate physical activity for ≥30 min, 5 days in the last week), obesity (body mass index of ≥30 kg/m2), hypertension (systolic blood pressure of ≥140 mmHg, diastolic blood pressure of ≥90 mmHg, or hypertension medication), and diabetes (fasting blood sugar level of ≥126 mg/dL or diabetes medication).

### 2.3. Statistical Analysis

The prevalence of osteoporosis and rate of diagnosis for each independent variable category were calculated using a univariate analysis, and the χ2 test was performed to determine the statistical significance. In addition, the prevalence and diagnosis rate of osteoporosis among the basic living beneficiaries and non-beneficiaries were calculated for each residential area, and statistical significance between the two was determined using the χ2 test. Multivariate logistic regression analyses were performed to identify the associations between the factors after adjusting for other factors. In addition, multivariate logistic regression analyses stratified into rural and urban areas were performed to determine the association between basic livelihood conditions and osteoporosis prevalence and diagnosis. Statistical significance was set at *p*-value of <0.05. All statistical analyses were performed using STATA MP 15.1 (Stata Corporation, College Station, TX, USA).

## 3. Results

### 3.1. General Characteristics of the Study Population

Table 1 presents the baseline characteristics of the participants. Women aged 50–59 years were the most common subgroup population (37.4%), followed by those aged 60–69 and ≥70 years. Women who graduated from junior high school or lower formed most of the sample (80.1%). The proportion of basic living recipients was 8.2%. In addition, 20.1% of the participants were categorized into a group engaging in physical activity. The prevalence of hypertension and diabetes was 51.2% and 13.5%, respectively. The proportion of the samples living in urban and rural areas was 70.6% and 29.4%, respectively. The proportion of older or less educated groups of subjects living in rural areas was significantly higher than that of subjects living in urban areas (*p* < 0.001). In particular, the proportion of basic livelihood beneficiaries in the samples living in rural areas was 12.7%, which was significantly higher than that in the samples living in urban areas (6.3%) (*p* < 0.001).

### 3.2. Osteoporosis Prevalence and Diagnosis

Table 2 shows the prevalence and diagnosis rate of osteoporosis according to factors. The overall prevalence and diagnosis rates were 34.8% and 22.0%, respectively. Both the prevalence and diagnosis rates were significantly higher in the older and less educated groups. The prevalence of osteoporosis was higher in the patients with hypertension and physical inactivity than in the other patients. In addition, the prevalence was higher in the basic livelihood beneficiaries and rural area residents than in the non-beneficiaries and urban area residents. Conversely, there was no significant difference in the diagnosis rates in relation to the basic livelihood conditions and residence areas.

**Figure 1:**
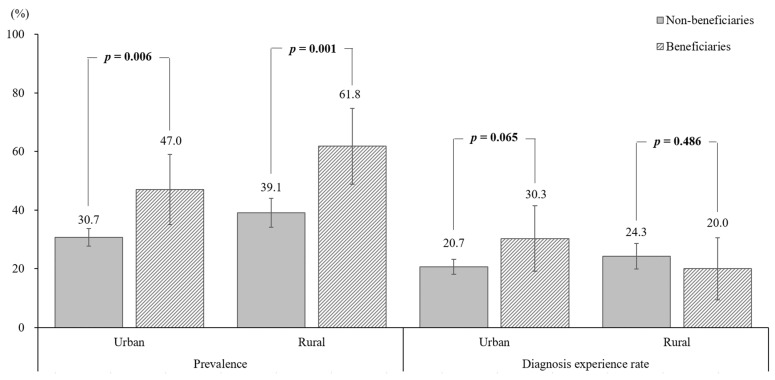
Osteoporosis prevalence and diagnosis experience rates by basic livelihood condition according to the areas of residence.

Figure 1 presents the prevalence and diagnosis rate of osteoporosis according to basic livelihood conditions between the urban and rural areas. In both urban and rural areas, the prevalence among the basic livelihood beneficiaries was 47.0% (95% confidence interval of 34.6~59.7) and 61.8% (95% confidence interval of 47.7~74.6), respectively, which was significantly higher than that among the non-beneficiaries (urban 30.7%, 95% confidence interval of 27.9~33.7) (rural 39.1%, 95% confidence interval 34.1~44.2). Meanwhile, the diagnosis rate was not significantly different.

### 3.3. Influence of the Factors on the Osteoporosis Prevalence and Diagnosis Rate in the Urban and Rural Areas

Table 3 shows the influence of the factors on the osteoporosis prevalence and diagnosis rate in all subjects. The factors that showed a significant impact on the prevalence were the age group, educational level, obesity, diabetes, and residence area. The adjusted odds ratios for the osteoporosis prevalence were significantly higher in the elderly (OR 8.83, *p* < 0.001 in those aged ≥70 years) and middle school or below categories (OR 2.47, *p* < 0.001) than in the other categories. Meanwhile, the risk of osteoporosis was significantly lower in the patients with obesity (OR 0.33, *p* = 0.001) and diabetes (OR 0.54, *p* = 0.001). In addition, residence in rural areas was associated with a significantly higher risk of osteoporosis than residence in urban areas (OR 1.41, *p* = 0.001). However, the only factor that significantly affected the diagnosis rate was age.

### 3.4. Influence of the Basic Livelihood Condition on the Osteoporosis Prevalence and Diagnosis Rate in the Rural and Urban Areas

Table 4 shows the influence of the basic livelihood condition on the osteoporosis prevalence and diagnosis rate by residence area. In the urban areas, the basic livelihood conditions were not significant factors for both prevalence and diagnosis rate after adjusting for the impacts of other factors (Model 1 and Model 2). However, in the rural areas, the odds ratio for the prevalence among the basic livelihood beneficiaries was 2.08 (95% confidence interval of 1.06~4.10, *p* = 0.033), which was significantly higher than that among the non-beneficiaries (Model 3). The influence of the basic livelihood conditions on the diagnosis rate was not significantly different, similar to the results for the urban areas.

## 4. Discussion

This study is the first to identify and compare the prevalence and diagnosis rate of osteoporosis between urban and rural areas and the influence of socioeconomic factors. In our analysis, the residents of rural areas and basic livelihood beneficiaries had a higher prevalence of osteoporosis than the residents of urban areas and non-beneficiaries; however, the diagnosis rate was not significantly different. In particular, in the analysis controlled for the influencing factors, the risk of osteoporosis was significantly higher among the basic livelihood beneficiaries in rural areas; however, the risk of diagnosis did not show a significant difference.

Many studies have investigated the risk factors of osteoporosis. Among these, age is considered the greatest risk factor [33,34,35]. In this study, both the prevalence and diagnosis rate of osteoporosis increased with age in all urban and rural areas. However, the adjusted odds ratio for the osteoporosis prevalence in the patients aged 60–69 years and ≥70 years was 2.62 and 8.83, respectively; meanwhile, the adjusted odds ratio for the diagnosis rate in the patients aged 60–69 years and ≥70 years was 2.81 and 4.33, respectively. These results indicate that osteoporosis is not detected early and that there are many undiagnosed elderly patients.

In the survey of the prevalence of osteoporosis, some studies have shown that the prevalence is higher in rural areas than in urban areas [14,36]. In particular, the proportion of women in households with economic difficulties is high [37]. Our study showed results similar to those of previous studies. However, there was no difference in the rate of diagnosis between the urban and rural areas. This means that diagnostic tests were not conducted in rural areas with a high prevalence. Ewald et al. reported that three-fold more osteoporosis tests were performed in urban areas than in rural areas [38]. In addition, Lai et al. reported that there were many undertreated patients in rural areas, and the prescription rate of osteoporosis drugs was low therein [16]. There are studies showing that there is a difference in the prevalence between urban and rural areas in chronic diseases such as cancer, diabetes, and kidney disease as well as osteoporosis [18,19,20]. In Han’s study on the rate of national screening, it was found that urban residents were 1.42 times more likely to receive health screenings than rural residents. In this study, it was reported that, for this reason, it is difficult for rural residents to access health examination institutions, and so the screening examination rate is low [21]. Additionally, in Vanasse’s study, access to professional services in non-metropolitan areas was significantly lower for myocardial infarction, osteoporosis, and diabetes [22]. In view of these results, chronic diseases such as osteoporosis are related not only to individual factors but also to various socioeconomic factors. Therefore, it is necessary to develop a screening service suitable for rural areas and find easily available national policies.

In our study, the prevalence of osteoporosis was high among the basic livelihood beneficiaries in rural areas; however, there was no difference in the diagnosis rate. With respect to low economic income levels, Korea is also implementing policies for basic livelihood beneficiaries. However, these basic recipients may have less access to medical care in hospitals than patients with private insurance [39,40]. In the case of basic recipients, there was also a report that as the level of education was low, the awareness of health was also low, and that they could neglect healthcare due to economic conditions [21]. Furthermore, in Korea, basic recipients must pay some costs for osteoporosis tests. If the osteoporosis test is not covered by health insurance, they must pay a greater amount, which yields a huge financial burden. Therefore, active prevention policies to prevent osteoporosis should be implemented for women aged over 50 years living in rural areas where medical access is difficult, especially basic livelihood beneficiaries who have economic difficulties.

In the multivariate logistic regression analysis of the risk factors for the prevalence of osteoporosis in our study, there were differences found in the educational level, obesity, and diabetes. Reid reported that obesity has many mechanisms for the adipose–bone relationship that can reduce osteoporosis [41]. These include the effect of soft tissue mass on skeletal load, the amount of bone-activating hormone and fat in pancreatic beta cells (e.g., insulin, amylin, and insulin), and secretion of bone-activating hormones (e.g., estrogen and leptin) from fat cells. In the study by Vestergaard [42], the hip fracture risk was reported to have increased in both type 1 and type 2 diabetes, whereas the BMD increased in type 2 diabetes and decreased in type 1 diabetes; however, the mechanism of the relationship between diabetes and BMD requires further study.

Our study has several limitations. The first was the cross-sectional nature of the study and the small sample size. Second, smoking and drinking history were not included as factors in the study. This is because the smoking rate is very low in the rural population, and statistical stability was not secured in the multivariate analysis. Third, men were not included. Although the prevalence of osteoporosis is low in men, future research will need to be conducted on the relationship between osteoporosis and socioeconomic factors including men as a study group. Fourth, hematological tests were not included in the examination for osteoporosis. Lastly, further prospective cohort studies, long-term follow-up studies, and studies on the fracture risk of patients with osteoporosis are needed.

## 5. Conclusions

The risk of osteoporosis was higher in the elderly subjects and basic living beneficiaries living in rural areas. The prevalence of osteoporosis among rural residents was higher than that among city dwellers. However, the osteoporosis diagnosis rate between the rural and urban areas was not significantly different. This indicates that there are many patients with undiagnosed osteoporosis. Therefore, it is necessary to establish a policy and conduct interventions for the early diagnosis of osteoporosis in a population with a low socioeconomic status living in rural areas. In addition, it is necessary to provide physical activity programs and medication for the prevention of osteoporosis.

## Figures and Tables

**Table 1:** General characteristics of the study population.

Variables		Total		Urban		Rural		*p*-Values ^a^
		n	%	n	%	n	%	
Total		1476	100.0	1042	100.0	434	100.0	
Age group (year)	50–59	552	37.4	420	40.3	132	30.4	<0.001
	60–69	505	34.2	364	34.9	141	32.5	
	≥70	419	28.4	258	24.8	161	37.1	
Educational level	≤Middle school	1183	80.1	781	75.0	402	92.6	<0.001
	≥High school	293	19.9	261	25.0	32	7.4	
Basic livelihood condition	Non-beneficiaries	1355	91.8	976	93.7	379	87.3	<0.001
	Beneficiaries	121	8.2	66	6.3	55	12.7	
Physical activity ^b^	No	1179	79.9	850	81.6	329	75.8	0.012
	Yes	297	20.1	192	18.4	105	24.2	
Obesity ^c^	No	1406	95.3	998	95.8	408	94.0	0.145
	Yes	70	4.7	44	4.2	26	6.0	
Hypertension ^d^	No	720	48.8	520	49.9	200	46.1	0.181
	Yes	756	51.2	522	50.1	234	53.9	
Diabetes ^e^	No	1277	86.5	890	85.4	387	89.2	0.054
	Yes	199	13.5	152	14.6	47	10.8	
Residence area	Urban	1042	70.6					
	Rural	434	29.4					

^a^ Results of the χ^2^ test between the urban and rural areas. ^b^ Engaging in vigorous physical activity for >20 min, 3 times a week or moderate physical activity for >30 min, 5 days in the last week. ^c^ Body mass index of >30 kg/m^2^. ^d^ Systolic blood pressure of >140 mmHg, diastolic blood pressure of >90 mmHg, or hypertension medication. ^e^ Fasting blood sugar level of >126 mg/dL or diabetes medication.

**Table 2:** Osteoporosis prevalence and diagnosis rates according to factors.

Variables		Prevalence			Diagnosis		
		n	%	χ^2^ Test *p*-Values	n	%	χ^2^ Test *p*-Values
Total		513	34.8		325	22.0	
Age group (year)	50–59	82	14.9	<0.001	61	11.1	<0.001
	60–69	165	32.7		126	25.0	
	≥70	266	63.5		138	32.9	
Educational level	≤Middle school	472	39.9	<0.001	325	23.2	0.033
	≥High school	41	14.0		51	17.4	
Basic livelihood condition	Non-beneficiaries	448	33.1	<0.001	294	21.9	0.318
	Beneficiaries	65	53.7		31	25.6	
Physical activity	No	426	36.1	0.027	252	21.4	0.234
	Yes	87	29.3		73	24.6	
Obesity	No	500	35.6	0.004	314	22.3	0.192
	Yes	13	18.6		11	15.7	
Hypertension	No	224	31.1	0.004	151	21.0	0.344
	Yes	289	38.2		174	23.0	
Diabetes	No	453	35.5	0.142	272	21.3	0.091
	Yes	60	30.2		53	26.6	
Residence area	Urban	331	31.8	<0.001	222	21.3	0.305
	Rural	182	41.9		103	23.7	

**Table 3:** Multivariate logistic regression analysis of the influence of the factors on the osteoporosis prevalence and diagnosis rate in the total population.

Variables		Prevalence			Diagnosis Rate		
		Adjusted OR	95% CI	*p*-Values	Adjusted OR	95% CI	*p*-Values
Age group (year)	50–59	Ref.			Ref.		
	60–69	2.62	(1.92–3.57)	<0.001	2.81	(1.99–3.98)	<0.001
	≥70	8.83	(6.33–12.32)	<0.001	4.33	(3.00–6.24)	<0.001
Educational level	≥High school	Ref.			Ref.		
	≤Middle school	2.47	(1.69–3.62)	<0.001	0.92	(0.64–1.32)	0.637
Basic livelihood condition	Non-beneficiaries	Ref.			Ref.		
	Beneficiaries	1.51	(0.99–2.30)	0.057	0.96	(0.61–1.50)	0.847
Physical activity	No	Ref.			Ref.		
	Yes	0.77	(0.56–1.04)	0.091	1.33	(0.97–1.81)	0.072
Obesity	No	Ref.			Ref.		
	Yes	0.33	(0.17–0.63)	0.001	0.60	(0.31–1.18)	0.139
Hypertension	No	Ref.			Ref.		
	Yes	0.92	(0.72–1.19)	0.529	0.86	(0.66–1.12)	0.269
Diabetes	No	Ref.			Ref.		
	Yes	0.54	(0.37–0.77)	0.001	1.17	(0.82–1.67)	0.396
Residence area	Urban	Ref.			Ref.		
	Rural	1.41	(1.11–1.71)	0.001	1.01	(0.76–1.34)	0.927

Odds ratio adjusted for all other confounders (age, education level, basic livelihood condition, physical activity, obesity, hypertension, diabetes, residence area). CI, confidence interval.

**Table 4:** Multivariate logistic regression analysis of the influence of the basic livelihood condition on the osteoporosis prevalence and diagnosis rate in the rural and urban areas.

Models ^a^	Residence Area	Dependent Variables	Independent Variable (Basic Livelihood Condition)	Adjusted OR	95% CI	*p*-Values
Model 1	Urban	Prevalence	Non-beneficiaries	Ref.		
			Beneficiaries	1.24	(0.71–2.17)	0.447
Model 2		Diagnosis rate	Non-beneficiaries	Ref.		
			Beneficiaries	1.34	(0.76–2.37)	0.305
Model 3	Rural	Prevalence	Non-beneficiaries	Ref.		
			Beneficiaries	2.08	(1.06–4.10)	0.033
Model 4		Diagnosis rate	Non-beneficiaries	Ref.		
			Beneficiaries	0.56	(0.27–1.17)	0.120

^a^ All models were adjusted for age group, educational level, physical activity, obesity, hypertension, and diabetes. OR, odds ratio; CI, confidence interval.

## Data Availability

All data are available from the database of the Korea National Health and Nutrition Examination Survey (https://knhanes.cdc.go.kr/ (accessed on 5 June 2021)).
